# MARCH8 Targets Cytoplasmic Lysine Residues of Various Viral Envelope Glycoproteins

**DOI:** 10.1128/spectrum.00618-21

**Published:** 2022-01-12

**Authors:** Yanzhao Zhang, Seiya Ozono, Takuya Tada, Minoru Tobiume, Masanori Kameoka, Satoshi Kishigami, Hideaki Fujita, Kenzo Tokunaga

**Affiliations:** a Department of Pathology, National Institute of Infectious Diseases, Tokyo, Japan; b Faculty of Life and Environmental Sciences, University of Yamanashi, Yamanashi, Japan; c Department of Microbiology, New York University School of Medicine, New York, New York, USA; d Department of Public Health, Kobe University Graduate School of Health Sciences, Hyogo, Japan; e Faculty of Pharmaceutical Sciences, Nagasaki International University, Nagasaki, Japan; Regional Centre for Biotechnology

**Keywords:** MARCH8, viral envelope glycoprotein, downregulation, ubiquitination, degradation

## Abstract

The host transmembrane protein MARCH8 is a RING finger E3 ubiquitin ligase that downregulates various host transmembrane proteins, such as MHC-II. We have recently reported that MARCH8 expression in virus-producing cells impairs viral infectivity by reducing virion incorporation of not only HIV-1 envelope glycoprotein but also vesicular stomatitis virus G-glycoprotein through two different pathways. However, the MARCH8 inhibition spectrum remains largely unknown. Here, we show the antiviral spectrum of MARCH8 using viruses pseudotyped with a variety of viral envelope glycoproteins. Infection experiments revealed that viral envelope glycoproteins derived from the rhabdovirus, arenavirus, coronavirus, and togavirus (alphavirus) families were sensitive to MARCH8-mediated inhibition. Lysine mutations at the cytoplasmic tails of rabies virus-G, lymphocytic choriomeningitis virus glycoproteins, SARS-CoV and SARS-CoV-2 spike proteins, and Chikungunya virus and Ross River virus E2 proteins conferred resistance to MARCH8. Immunofluorescence showed impaired downregulation of the mutants of these viral envelope glycoproteins by MARCH8, followed by lysosomal degradation, suggesting that MARCH8-mediated ubiquitination leads to intracellular degradation of these envelopes. Indeed, rabies virus-G and Chikungunya virus E2 proteins proved to be clearly ubiquitinated. We conclude that MARCH8 has inhibitory activity on a variety of viral envelope glycoproteins whose cytoplasmic lysine residues are targeted by this antiviral factor.

**IMPORTANCE** A member of the MARCH E3 ubiquitin ligase family, MARCH8, downregulates many different kinds of host transmembrane proteins, resulting in the regulation of cellular homeostasis. On the other hands, MARCH8 acts as an antiviral factor when it binds to and downregulates HIV-1 envelope glycoprotein and vesicular stomatitis virus G-glycoprotein that are viral transmembrane proteins. This study reveals that, as in the case of cellular membrane proteins, MARCH8 shows broad-spectrum inhibition against various viral envelope glycoproteins by recognizing their cytoplasmic lysine residues, resulting in lysosomal degradation.

## INTRODUCTION

Membrane-associated RING-CH (MARCH) 8 is one of 11 MARCH family members of RING-finger E3 ubiquitin ligases and consists of a short ectodomain connecting two transmembrane domains with two cytoplasmic amino and carboxyterminal domains (1, 2). MARCH8 is known to downregulate various types of host transmembrane proteins, including MHC-II (3, 4), CD86 (5, 6), CD81 (7), CD44 (7, 8), TRAIL receptor 1 (9), CD98 (8, 10), Bap31 (7), IL-1 receptor accessory protein (11), transferrin receptor (12), and cadherin-1 (13). We have recently reported that MARCH8 downregulates HIV-1 envelope glycoprotein (Env) from the cell surface, thereby reducing the infectivity of viruses produced from MARCH8-expressing cells (14). Importantly, the antiviral effect of MARCH8 was observed in not only HIV-1 Env but also vesicular stomatitis virus G-glycoprotein (VSV-G), which was even more sensitive to this host protein (14). Furthermore, we have recently shown that MARCH8 inhibits VSV-G-or HIV-1 Env-mediated viral infectivity by two different mechanisms through ubiquitination-dependent or tyrosine motif-dependent pathways, respectively (15). Given that these two viral envelope glycoproteins are structurally and phylogenetically unrelated to each other, we conjecture that MARCH8 might show broad-spectrum inhibition of enveloped viruses by downregulating viral envelope glycoproteins, exactly as reported for the aforementioned host membrane proteins. Here, we show that MARCH8 can target a variety of viral envelope glycoproteins by performing pseudotyping assays, consistent with recently published reports (16–18).

## Results

### MARCH8 expression in producer cells inhibits infection by pseudoviruses harboring various viral envelope glycoproteins.

**FIG 1:**
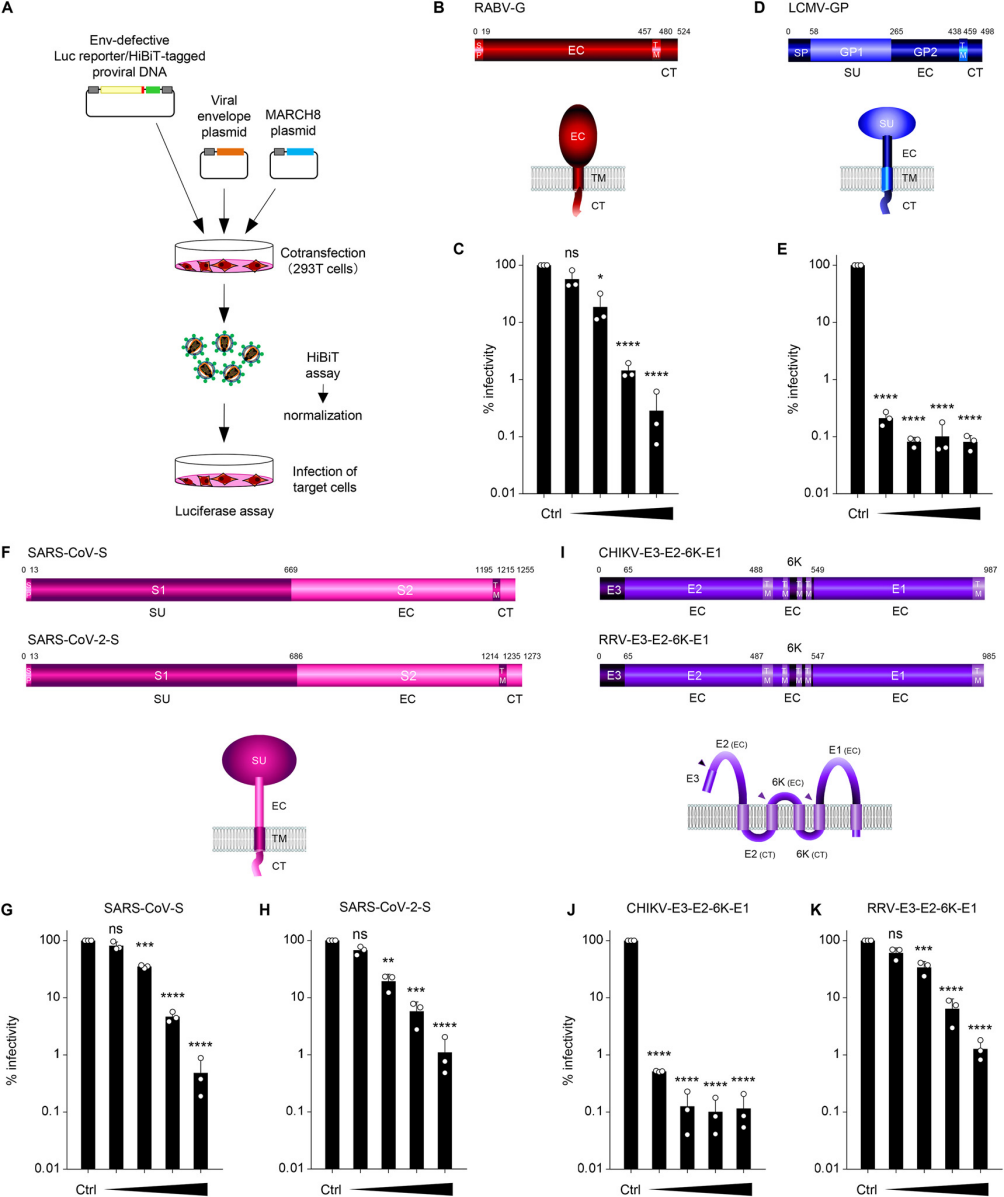
MARCH8 expression in producer cells decreases the infectivity of viruses pseudotyped with a variety of viral envelope glycoproteins. (A) Schematic flowchart of the experimental procedure for pseudovirus infectivity assays. Pseudoviruses were produced from 293T cells cotransfected with Env-defective HiBiT-tagged HIV-1 luciferase (luc) reporter proviral DNA clone pNL-Luc2-IN/HiBiT-E(-)Fin and either a control (Ctrl) or increasing amounts of the MARCH8 plasmid (30, 60, 120, and 240 ng) together with various viral envelope plasmids. Produced viruses were subjected to HiBiT assays to determine the level of virion production. Equivalent amounts of p24 antigen (translated from HiBiT-luc activities) of pseudoviruses were used for the infection of different target cells, as described below. After 48 h, cells were lysed, and firefly luc activities were measured to determine viral infectivity. (B, D, F, and I) Schematic gene structures and topology of the various viral envelope glycoproteins. (C, E, G, H, J, and K) Various viral envelope glycoproteins tested are differentially susceptible to MARCH8-mediated inhibition. Viral infectivity was determined from infection using viruses pseudotyped with (C) rabies virus G (RABV-G) in 293T cells; (E) lymphocytic choriomeningitis virus (LCMV) envelope GP in NIH 3T3 cells; (G) severe acute respiratory syndrome-associated coronavirus (SARS-CoV) and (H) SARS-CoV-2 spike in 293T cells coexpressing ACE2 receptor and the transmembrane protease TMPRSS2; and (J, K) alphavirus E3-E2-6K-E1 (from (J) Chikungunya virus (CHIKV) and (K) Ross River virus (RRV)) in HeLa cells. Data from three independent experiments are shown as a percentage of the infectivity of viruses produced without MARCH8 (mean ± s.d., *n *= 3 technical replicates). *P < 0.05, **P < 0.005, ***P < 0.0005, ****P < 0.0001 compared with the Ctrl using two-tailed unpaired *t*-tests. ns, not significant. Schematic representations of genes and topological structures illustrating viral envelope glycoproteins are shown in the upper and middle panels. SP, signal peptide; EC, extracellular domain; TM, transmembrane domain; CT, cytoplasmic tail; SU, surface subunit. Arrowheads in the middle panel of Fig. 1I represent cleavage sites of alphavirus E3-E2-6K-E1.

To investigate the antiviral spectrum of MARCH8, we used luciferase-reporter/HiBiT-tagged HIV-1 (19) pseudotyped with a variety of viral envelope glycoproteins (Fig. 1A). Because VSV, whose glycoprotein is highly susceptible to MARCH8-mediated inhibition (14), belongs to the rhabdovirus family, we first tested another rhabdovirus-derived envelope, rabies virus (RABV)-G (Fig. 1B). Viral infectivity assays showed that this virus was sensitive to MARCH8 in a dose-dependent manner (Fig. 1C). We next tested the prototypic arenavirus lymphocytic choriomeningitis virus (LCMV) envelope GP, which carries a single transmembrane domain (Fig. 1D), along with the aforementioned viral envelope glycoproteins. LCMV-GP was found to be particularly sensitive, even in the presence of only a small amount of MARCH8 expression plasmid (Fig. 1E). Other viral envelope glycoproteins with a single transmembrane domain, i.e., severe acute respiratory syndrome-associated coronavirus (SARS-CoV) and SARS-CoV-2 spike (S) proteins (Fig. 1F), were also inhibited by MARCH8 in a dose dependent manner (Fig. 1G and H). In infection by pseudoviruses with alphavirus envelopes that are initially expressed as polyproteins with multiple transmembrane domains (Fig. 1I), Chikungunya virus (CHIKV) envelope was found to be especially highly susceptible to MARCH8 (Fig. 1J), whereas the Ross River virus (RRV) envelope, was also dose-dependently inhibited (Fig. 1K). Consistent with the impaired infectivity of these pseudoviruses, the levels of all viral envelope glycoproteins tested were remarkably reduced in virions that were produced from MARCH8-expressing cells (Fig. S1). Note that S proteins of SARS-CoV and SARS-CoV-2 showed not only reduced levels of virion incorporation but also decreased size of the Western blotting band probably due to a putatively less glycosylation, as reported by Lun et al. (17).

### MARCH8 targets cytoplasmic lysine residues of viral envelope glycoproteins with single transmembrane domains.

**FIG 2:**
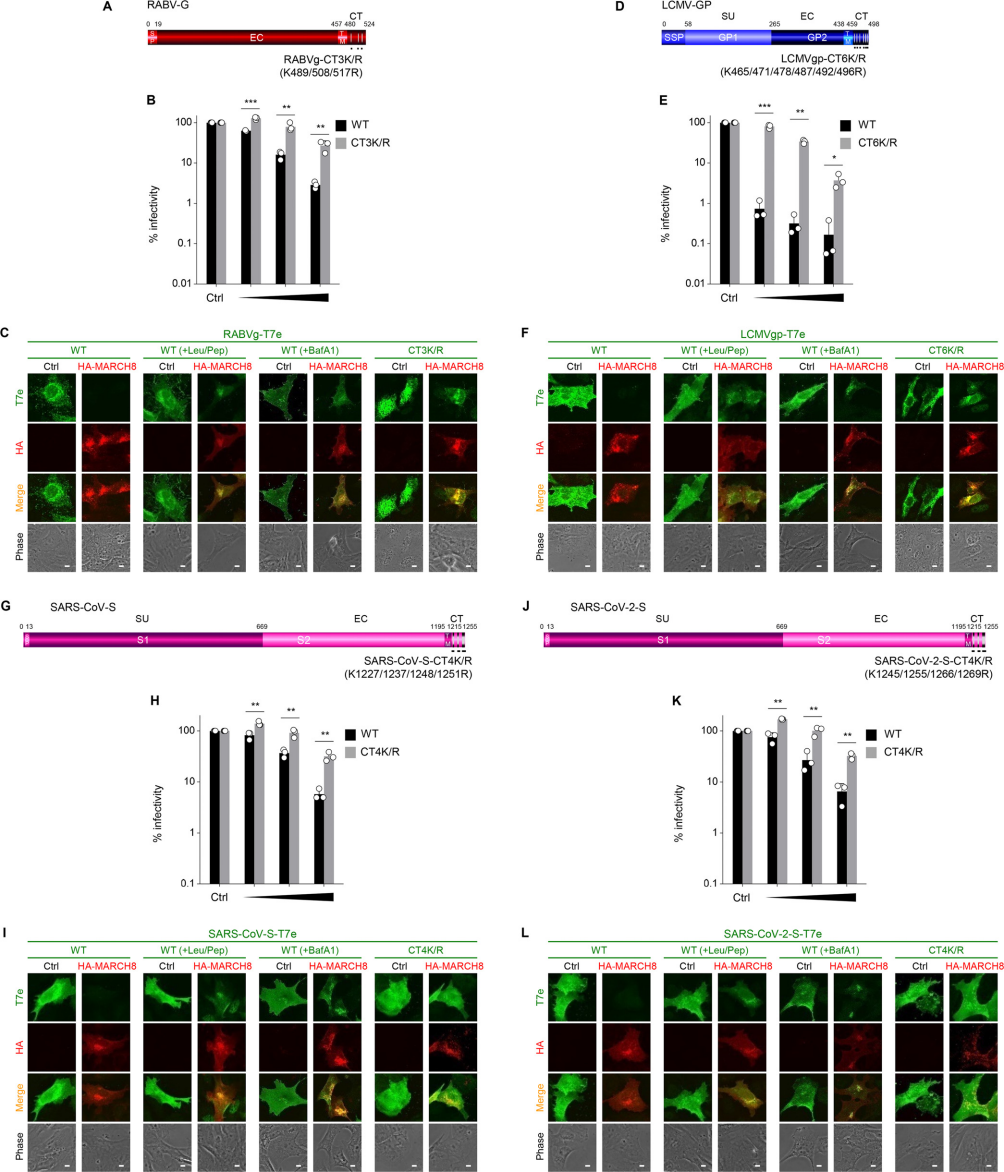
MARCH8 targets lysine residues in the cytoplasmic tail of viral envelope glycoproteins with single transmembrane domains. (A, D, G, and J) Schematic gene structures of the lysine mutants of (A) RABV-G (CT3K/R); (D) LCMV-GP (CT6K/R); (G) SARS-CoV-S (CT4K/R); and (J) SARS-CoV-2-S (CT4K/R). SP, signal peptide; EC, extracellular domain; TM, transmembrane domain; CT, cytoplasmic tail; SU, surface subunit. (B, E, H, and K) MARCH8 resistance is conferred by mutations in cytoplasmic lysine residues of viral envelope glycoproteins with single transmembrane domains. Infectivity assays were performed as described in Fig. 1A except that the maximum amount of MARCH8 plasmid used was 120 ng. Black and gray columns represent the wild-type (WT) and lysine mutants of each viral envelope glycoprotein, respectively. Data from three independent experiments are shown as a percentage of the infectivity of viruses produced in the absence of MARCH8 when the WT protein or its mutant was used (mean ± s.d., *n *= 3 technical replicates). *P < 0.05, **P < 0.005, ***P < 0.0005, ****P < 0.0001 compared with the WT using two-tailed unpaired *t*-tests. ns, not significant. (C, F, I, and L) Lysine mutants of viral envelope glycoproteins are resistant to MARCH8-mediated lysosomal degradation. Shown are immunofluorescence-based analyses of the expression of either the T7 epitope (T7e)-tagged WT or K/R mutant of (C) RABV-G; (F) LCMV-GP; (I) SARS-CoV-S; and (L) SARS-CoV-2-S with or without MARCH8 in transfected HOS cells. All WT viral envelope glycoproteins were rescued from MARCH8-induced degradation in the presence of lysosomal inhibitors (leupeptin/pepstatin (+Leu/Pep), or bafilomycin A1 (+BafA), as shown in each of the middle panels. Scale bars, 10 μm. Protein expression of the mutants was confirmed by immunoblotting (Fig. S2).

We have recently reported that the cytoplasmic lysine residues of VSV-G are targeted by MARCH8 for ubiquitination and degradation (14), which prompted us to address whether MARCH8-sensitive viral envelope glycoproteins also harbor cytoplasmic lysines that could be ubiquitination targets for MARCH8. Among the above viral envelope glycoproteins, a single-spanning membrane protein, RABV-G, harbors three lysine residues at positions 489, 508, and 517 in its cytoplasmic tail. Therefore, we mutated these residues to arginines in this viral envelope glycoprotein (K489/508/517R) (Fig. 2A) and confirmed protein expression of the mutant (Fig. S2). Infectivity assays to compare the WT and mutant envelopes showed that the lysine mutations in RABV-G conferred resistance to MARCH8 (Fig. 2B). The cytoplasmic tail of the single-spanning membrane protein LCMV-GP contains six lysine residues at positions 465, 471, 478, 487, 492, and 496, which we mutated to arginine residues (K465/471/478/487/492/496R) (Fig. 2D). The effect of the mutation in LCMV-GP was more drastic in that the mutant envelope was resistant to MARCH8 (Fig. 2E), probably due to the larger number of lysine residues in LCMV-GP than in RABV-G. Other single-spanning membrane proteins, SARS-CoV-S and SARS-CoV-2-S, which were MARCH8-sensitive in a dose-dependent manner (Fig. 1G and H), harbor four lysine residues in their cytoplasmic tails (at positions 1227, 1237, 1248, and 1251 in SARS-CoV-S and 1245, 1255, 1266, and 1269 in SARS-CoV-2-S) (Fig. 2G and J). When these lysines were mutated to arginines, both S mutant proteins displayed relative resistance to MARCH8 (Fig. 2H and K). Immunofluorescence confirmed that MARCH8, which induced lysosomal degradation of wild-type envelopes, was unable to downregulate all of these mutant envelopes from the cell surface (Fig. 2C, F, I and L, and S3). These results suggest that MARCH8 targets lysine residues located in the cytoplasmic tails of these viral envelope glycoproteins, leading to their lysosomal degradation that might result from ubiquitination, as previously observed for VSV-G (15).

### MARCH8 targets a cytoplasmic lysine residue in alphavirus E2 proteins.

**FIG 3:**
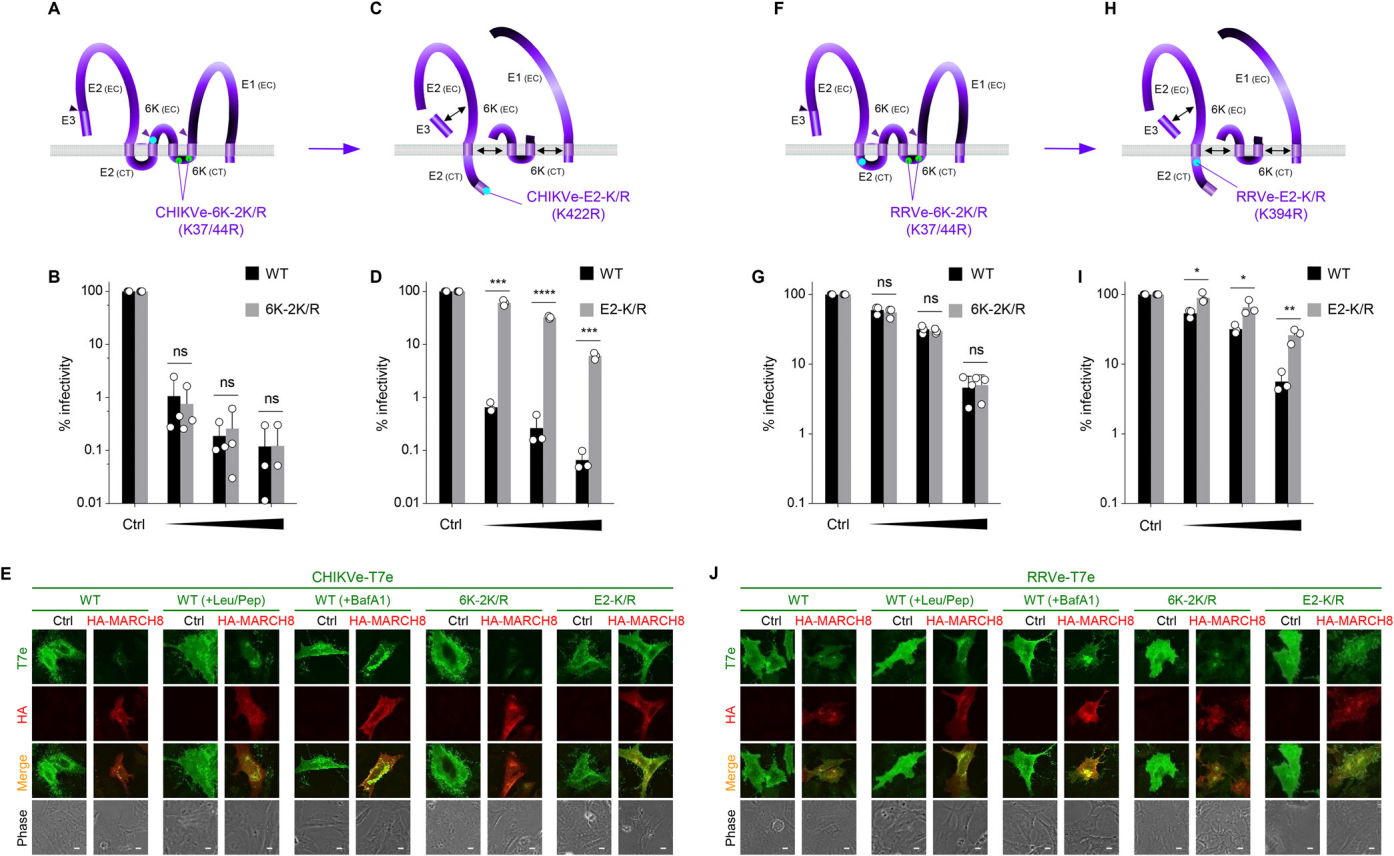
MARCH8 targets a cytoplasmic lysine residue in E2 but not in 6K of alphaviruses. (A, C, F, and H) Schematic representation of topological structures illustrating the multiple-spanning membrane proteins of CHIKV-E3-E2-6K-E1 (A, C) and RRV-E3-E2-6K-E1 (F, H). (A, F) In the precleavage state, two lysine residues (37K and 44K, shown in light green) of the 6K protein are exposed to the cytoplasm. These residues were mutated to arginine residues, and the resultant mutants were designated CHIKVe-6K-2K/R (A) and RRVe-6K-2K/R (F). Cleavage sites are shown by arrowheads. EC, extracellular domain; CT, cytoplasmic tail. (C, H) In the postcleavage state, a lysine residue (K422; light blue) at the C-terminal end of the CHIKV-E2 protein, which is present extracellularly in the precleavage state, as shown in Fig. 3A, is relocated and exposed to the cytoplasm (C). In contrast, a lysine residue of RRV-E2 (K394; light blue) was originally located at the proximal region of the internal membrane (H). Each lysine residue was replaced by an arginine, and the resultant mutants were designated CHIKVe-E2-K/R (C) and RRVe-E2-K/R (H). Double-headed arrows represent the putative interaction of cleaved viral proteins. (B, D, G, and I) MARCH8 resistance is conferred by the mutation of a cytoplasmic lysine residue of the alphavirus protein E2 (D, I) but not 6K (B, G). Infectivity assays were performed as described in Fig. 1A except that the maximum amount of MARCH8 plasmid used was 120 ng. Black and gray columns represent the WT and K/R mutants of alphaviruses, respectively. Data from three independent experiments are shown as a percentage of the infectivity of viruses produced in the absence of MARCH8 when the WT protein or its mutant was used (mean ± s.d., *n *= 3 technical replicates). *P < 0.05, **P < 0.005, ***P < 0.0005, ****P < 0.0001 compared with the Ctrl using two-tailed unpaired *t*-tests. ns, not significant. (E, J) Lysine mutants of alphavirus E2 but not 6K are resistant to MARCH8-mediated lysosomal degradation. Shown are immunofluorescence-based analyses of the expression of either the T7e-tagged WT or K/R mutants of CHIKV-E3-E2-6K-E1 (E) and RRV-E3-E2-6K-E1 (J) with or without MARCH8 in transfected HOS cells. Both WT viral envelope glycoproteins were rescued from MARCH8-induced degradation in the presence of lysosomal inhibitors (+Leu/Pep, or +BafA). Scale bars, 10 μm. Protein expression of the mutants was confirmed by immunoblotting (Fig. S2).

In the case of CHIKV and RRV polyproteins (E3-E2-6K-E1), cytoplasmic lysines are located in the 6K proteins (Fig. 3A and F); therefore, we introduced point mutations into the residues of these proteins and then performed infectivity assays. However, these mutants were found to still be sensitive to MARCH8 at exactly the same levels as the WT envelope (Fig. 3B and G), indicating that MARCH8 does not target the lysine residues in the 6K proteins of alphavirus envelope glycoproteins. After being proteolytically cleaved from 6K, the second transmembrane domain of the alphavirus E2 protein is released from the membrane and translocates along with its short ectodomain into the cytoplasm, after which the cleaved E2 protein is associated with E3, 6K, and E1 (20) (Fig. 3C and H). Based on this process, we noticed that K422 of CHIKV-E2 could be translocated from the membrane into the cytoplasm after E2 cleavage (Fig. 3C), whereas K394 of RRV-E2 was originally exposed in the cytoplasm (Fig. 3F and H), both of which were mutated to arginine (K422R and K394R, respectively). The K422R mutation rendered the CHIKV envelope resistant to MARCH8 (Fig. 3D), while the K394 mutation of RRV showed a modest but noticeable effect on MARCH8-mediated inhibition (Fig. 3I). The results were consistent with those of immunofluorescence showing that MARCH8-mediated lysosomal degradation observed in the WT envelope (which is C-terminally tagged with the T7 epitope [T7e]) was rescued in the E2-lysine mutants but not in the 6K mutants (Fig. 3E and J, and S3), suggesting that these two viral envelope glycoproteins are downregulated by MARCH8 in a manner dependent on E2 domain-specific ubiquitination.

### MARCH8 ubiquitinates lysine residue(s) at the CT domains of RABV-G and CHIKV-E2.

**FIG 4:**
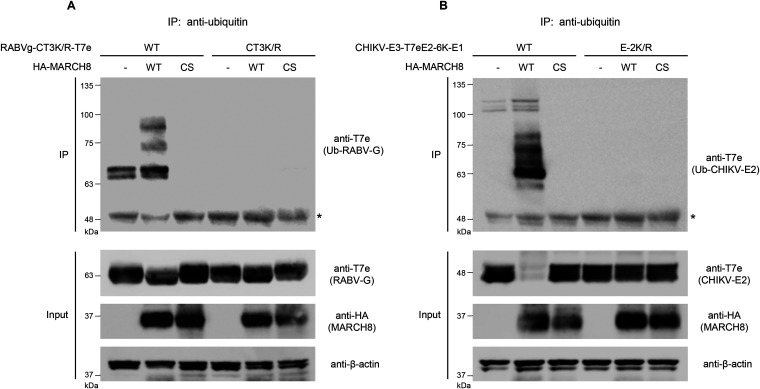
MARCH8, but not its RING-CH mutant, ubiquitinates cytoplasmic lysine residue(s) of RABV-G and CHIKV-E2. (A) The ubiquitination of the WT or CT-lysine mutant of RABV-G (CT3K/R) protein C-terminally tagged with T7e in cells expressing control (-), HA-tagged MARCH8 (WT), or its RING-CH mutant (CS), was examined by the immunoprecipitation (IP) of ubiquitinated proteins with an anti-ubiquitin antibody, followed by immunoblotting with an anti-T7e antibody. Aliquots of the cell lysates (Input) were also analyzed by immunoblotting for RABV-G (upper), MARCH8 (middle), and β-actin (lower). (B) The ubiquitination of the WT or E2-lysine mutant of CHIKV-E3-E2-6K-E1 (E2-K/R) protein, in which the E2 domain was tagged with T7e, was similarly investigated (*upper*). Lysates were also analyzed by immunoblotting for CHIKV-E2 (upper), MARCH8 (middle), and β-actin (lower). Note that, in the third lanes of IP in (A) and (B), ubiquitinated bands seen in the control lanes (MARCH8 [-]) disappear, probably due to the dominant negative effect of the MARCH8 mutant protein. Asterisks indicate the positions of immunoglobulin G heavy chains.

To demonstrate whether the aforementioned cytoplasmic lysine residues of MARCH8-sensitive viral envelope glycoproteins are targets for MARCH8-mediated ubiquitination, we performed immunoprecipitation/Western-based ubiquitination assays using cells coexpressing either control or MARCH8, together with either the RABV-G (which is C-terminally T7e-tagged) or the CHIKV envelope glycoprotein (in which a T7e-tag is inserted immediately downstream of a furin-cleavage site between E3 and E2, resulting in an N-terminally T7e-tagged E2). In cells coexpressing MARCH8, both RABV-G (Fig. 4A) and CHIKV-E2 (Fig. 4B) were efficiently ubiquitinated, whereas their lysine mutants (CT3K/R and E2-K/R, respectively) did not undergo MARCH8-mediated ubiquitination, as expected, suggesting that lysine residue(s) at either position 489, 508, and 517 in the CT domain of RABV-G or position 422 in E2 of CHIKV are specifically ubiquitinated by MARCH8. Taken together, these findings indicate that MARCH8 ubiquitinates cytoplasmic lysine residues of a variety of viral envelope glycoproteins and that this ubiquitination leads to lysosomal degradation.

### The tyrosine motif of MARCH8 is important for its antiviral activity against some viruses.

The aforementioned MARCH8-sensitive viral envelope glycoproteins could be considered ubiquitination-sensitive proteins due to the observation of lysine-dependent degradation by MARCH8. We also examined whether these viral proteins could undergo MARCH8 tyrosine motif-dependent downregulation, as previously observed in HIV-1 Env (15)). As expected, infection by viruses pseudotyped with LCMV-GP or CHIKV-E3-E2-6K-E1, which are highly sensitive to MARCH8 (Fig. 1E and J), was still inhibited by the MARCH8 AxxL mutant (Fig. S4), as observed in VSV-G that is extremely MARCH8-sensitive (14, 15). Conversely, RABV-G, SARS-CoV-S, SARS-CoV-2-S, and RRV-E3-E2-6K-E1, whose functions were dose-dependently abrogated by MARCH8, were almost completely resistant to the AxxL mutant, respectively (Fig. S4), suggesting that the inhibition of these viral envelope glycoproteins is simultaneously regulated by two mechanisms of MARCH8, which are both ubiquitination- and tyrosine motif-dependent downregulations.

### MARCH1 and MARCH2 differentially target viral envelope glycoproteins.

We have recently reported that among MARCH family members, MARCH1 and MARCH2 are also antiviral MARCH proteins that inhibit HIV-1 infection (21). Therefore, we examined whether these two MARCH proteins could similarly target various viral envelope glycoproteins, as observed in MARCH8 in this study. Both MARCH1 and MARCH2 were found to be able to differentially inhibit infection by pseudoviruses harboring RABV-G, LCMV-GP, CHIKV and RRV envelope glycoproteins but not the S proteins of SARS-CoV and SARS-CoV-2 although MARCH8 showed the highest inhibitory activity against all viral envelope glycoproteins tested (Fig. S5).

### Antiviral activity of MARCH8 is reproducible in infection with RABV.

Finally, because all the aforementioned experiments were performed using pseudotyped viruses, we conducted whole-virus-based infection experiments to verify the inhibitory effects of MARCH8 on not only pseudotyped viruses but also whole viruses. We first prepared cells stably expressing MARCH8, used the cells for infection with whole RABV, and then performed immunofluorescence assays using antibodies against two different RABV proteins, phosphoprotein P and envelope glycoprotein G (Fig. S6A). As expected, RABV-G protein was completely lost from the surface of MARCH8-expressing cells infected with rabies viruses, whereas the expression of RABV-P was not affected by MARCH8 expression (Fig. S6B). Thus, the aforementioned results obtained in pseudotyped viruses were demonstrated to be reproducible in whole-virus infections and were specific for MARCH8-mediated inhibition.

## DISCUSSION

In this study, we show that MARCH8 can target a broad spectrum of viral envelope glycoproteins. Among viral envelope glycoproteins that have single-spanning membrane regions, MARCH8-sensitive proteins include those derived from not only retroviruses and a rhabdovirus VSV, as we previously reported (14), but also another rhabdovirus RABV, an arenavirus LCMV, and sarbecoviruses (SARS-CoV/SARS-CoV-2). Independent of virus family, the levels of MARCH8 sensitivity might be determined, to some extent, by the number of lysine residues in their cytoplasmic tails (Fig. S7) that could be ubiquitinated by MARCH8 under the condition that lysines are optimally exposed at which the ubiquitin ligase is readily accessible. Indeed, among single-spanning viral envelope glycoproteins, RABV-G and SARS-CoV/SARS-CoV-2 S proteins, whose cytoplasmic tails harbors three and four lysines out of 44 and 39 amino acids, respectively (Fig. S7), are moderately MARCH8-sensitive (Fig. 1C and G, and 1H). In contrast, VSV-G and LCMV-GP, whose cytoplasmic tails are lysine-rich (5 out of 29 and 6 out of 39 amino acids, respectively; Fig. S7), are highly susceptible to MARCH8 ([14] and Fig. 1E) but are, in turn, resistant to this host antiviral protein when their lysine residues are mutated to arginines. It should be noted that HIV-1 Env carries only two lysines out of 151 amino acids in the cytoplasmic tail but is still MARCH8-sensitive when these lysines are mutated to arginines (15).

Other viral envelope glycoproteins sensitive to MARCH8 are those derived from alphaviruses (CHIKV and RRV), whose envelope glycoproteins are initially expressed as polyproteins with multiple transmembrane domains. These polyproteins are proteolytically cleaved, resulting in single-spanning membrane proteins formed with E2-E1 heterodimers (20). In the case of CHIKV-E3-E2-6K-E1, which is extremely sensitive to MARCH8, a lysine mutation in E2 conferred strong resistance to MARCH8, suggesting that the lysine residue at the C-terminal edge of cleaved E2 (Fig. 3C) can easily access MARCH8 in the cytoplasm and is therefore critical for MARCH8 sensitivity. Based on the fact that only a moderate effect was observed in a mutation of the lysine located in the membrane-proximal position of the RRV-E2 cytoplasmic tail (Fig. 3H), it is likely that not only the number but also the location of the lysine residues, which possibly affects the accessibility of MARCH8, may also determine the sensitivity to MARCH8.

Unexpectedly, in the absence of MARCH8, the luciferase values before individual normalization showed that the infectivity of the aforementioned cytoplasmic lysine mutants was higher than that of their wild-type envelopes to various degrees (1.5 to 3-fold higher in those of RABV, LCMV, SARS-CoV, and CHIKV; 5-fold higher in that of SARS-CoV-2), except for that of the RRV-6K mutant (Fig. S8). Because our previous findings demonstrated that the endogenous expression of MARCH8 in 293T cells (also used for virus production in this study) is extremely low (14), it is unlikely that these viral envelope glycoproteins harboring lysine mutations successfully evaded inhibition by the low levels of MARCH8 in this cell line. Therefore, we postulate that unknown E3 ubiquitin ligase(s) endogenously expressed in 293T cells might be partially involved in targeting the lysine residues in the cytoplasmic tails of viral envelope glycoproteins. In fact, ubiquitination assays showed that WT RABV-G and CHIKV-E2 (but not their mutant) proteins were slightly ubiquitinated even in the absence of MARCH8 in 293T cells (Fig. 4), supporting the above hypothesis. Further investigations are needed to identify such unknown E3 ligases.

Among the MARCH8-sensitive viral envelope glycoproteins tested in this study, phenotypes of viral envelope glycoproteins are divided into two groups: (i) highly MARCH8-sensitive in the WT envelope and resistant in its lysine mutant (VSV-G, LCMV-GP, and CHIKV-E3-E2-6K-E1); and (ii) dose-dependently MARCH8-sensitive in the WT envelope and partially resistant in its lysine mutant (RABV-G, SARS-CoV-S, SARS-CoV-2-S, and RRV-E3-E2-6K-E1). In both types, MARCH8 clearly targets the cytoplasmic lysine residues of the viral envelope glycoproteins. Interestingly, the difference in WT MARCH8 sensitivity paralleled that in the resistance to the MARCH8 Yxxϕ mutant; i.e., whereas viral envelope glycoproteins in the case of (i) were still sensitive to the Yxxϕ mutant, those in the cases of (ii) were resistant to this mutant. These results suggest that MARCH8 downregulates (a) VSV-G, LCMV-GP, and CHIKV-E3-E2-6K-E1 in a ubiquitination-dependent pathway (Fig. S9, left) and (b) RABV-G, SARS-CoV-S, SARS-CoV-2-S, and RRV-E3-E2-6K-E1 in both ubiquitination- and Yxxϕ motif-dependent pathways (Fig. S9, middle). As an exceptional case compared with all other viral envelope glycoproteins tested here, it is intriguing that MARCH8-induced downregulation of HIV-1 Env is ubiquitination-independent and Yxxϕ motif-dependent, as we recently reported (15) (Fig. S9, right), and the ubiquitination independency is indeed consistent with a very recent paper (17) showing that MARCH8-mediated restriction of HIV-1 Env was independent of the presence of its cytoplasmic tail, which carries two lysine residues.

We acknowledge some limitations of the present study. Mainly, we performed extensive infection experiments using viruses pseudotyped with several different envelope glycoproteins, which might be physiologically different from bona fide infections by enveloped viruses and conducted whole-virus-based infection experiments using an RABV strain only. However, such pseudovirus experiments can still provide mechanistic insights underlying MARCH8-mediated inhibition of enveloped virus infection because MARCH8 intrinsically targets cellular or viral transmembrane proteins, which, in the case of enveloped viruses, are generally envelope glycoproteins. Indeed, our study demonstrates that MARCH8 downregulates various viral glycoproteins by targeting their cytoplasmic lysine residues leading to lysosomal degradation. Some of viral glycoproteins we tested here, such as SARS-CoV-2, LCMV, and CHIKV. have recently been identified as MARCH8 targets in a recent report (16), in which they showed that MARCH8 reduces the intracellular expression levels of these envelope glycoproteins without clarifying target residues and degradation pathway(s). In addition, we were only able to show MARCH8-mediated ubiquitination of RABV-G and CHIKV-E2, due to technical difficulties in detecting specific ubiquitinated/non-ubiquitinated bands obscured by high background signals in our ubiquitination assays. Further experiments will be required to verify the reproducibility of ubiquitination in other viral envelope glycoproteins.

During the preparation of the manuscript, three reports described above (16–18) showed that in addition to HIV-1 Env and VSV-G, MARCH8 has broad antiviral functions targeting viral glycoproteins from influenza virus, Ebola virus, murine leukemia virus, and Nipah virus (along with those from SARS-CoV-2, LCMV, and CHIKV, as described above). Yu et al. described that MARCH8 inhibits proteolytic cleavage of HIV-1 Env, Ebola virus GP (also reported by Lun et al. [17]), and influenza virus hemagglutinin, and that it also blocks glycosylation of Ebola virus GP in the Golgi, which results in the inhibition of its plasma membrane transport (18). In the case of SARS-CoV-2, Lun et al. showed that its lysine residues do not affect sensitivity to MARCH8, probably due to the results obtained from single-dose experiments (17). Nevertheless, the current consensus on the function of MARCH8 from these multiple studies, including ours, is that MARCH8 has a broad antiviral spectrum against various viral envelope glycoproteins, as we originally expected, and here, we conclude that MARCH8 targets the cytoplasmic lysine residues of these viral envelope glycoproteins. Further investigation will be needed to understand the broad antiviral mechanisms of this protein in more detail.

## MATERIALS AND METHODS

### Plasmids.

The envelope glycoprotein (Env)-deficient HiBiT-tagged HIV-1 proviral indicator construct pNL-Luc2-IN/HiBiT-E(-)Fin, the HIV-1 Gag-Pol expression plasmid pC-GagPol-RRE, the HIV-1 Rev expression plasmid pCa-Rev, the MARCH8 expression plasmid pC-MARCH8, pC-HA-MARCH8, and the tyrosine motif-mutant pC-MARCH8-^222^AxxL^225^, the MARCH1 expression plasmid pC-MARCH1, the MARCH2 expression plasmid pC-MARCH2, the severe acute respiratory syndrome coronavirus (SARS-CoV) spike (S) protein expression plasmid pC-SARS-S, the SARS-CoV-2-S protein expression plasmid pC-SARS2-S, the ACE2 expression plasmid pC-ACE2 and the TMPRSS2 expression plasmid pC-TMPRSS2, have previously been described elsewhere (14, 15, 19, 21–23). The plasmid pC-RABVg expressing the glycoprotein (G) of RABV was created by inserting the Acc65I/XhoI-digested G fragments (PCR-amplified from the RABV-G expression plasmid pzC26-G (24) derived from CVS rabies strain-infected cells (25)) into the corresponding site of pCAGGS. The plasmid pC-LCMVgp expressing the viral envelope glycoprotein derived from lymphocytic choriomeningitis virus (LCMV) glycoprotein (gp) was created by inserting PCR-amplified and BsiWI/XhoI-digested LCMV-GP fragments (PCR-amplified from pCI-LCMV-GP (26)) into the Acc65I/XhoI-digested pCAGGS mammalian expression plasmid. The plasmid pC-CHIKVe expressing alphavirus E3-E2-6K-E1 polyprotein derived from Chikungunya virus (CHIKV) was created by inserting the PCR-amplified and Acc65I/XhoI-digested E3-E2-6K-E1 fragments (PCR-amplified from codon-optimized CHIKV’s C-E3-E2-6K-E1 plasmid (27)) into the corresponding site of pCAGGS. Another E3-E2-6K-E1 expression plasmid, pC-RRVe, derived from the Ross River virus (RRV) T48 strain, was created by inserting the PCR-amplified and EcoRV/NotI-digested E3-E2-6K-E1 fragments (PCR-amplified from pRRV-E2E1 (28)) into the corresponding site of pCAGGS. The RABV-G or LCMV-GP mutant (pC-RABVg-CT3K/R, or pC-LCMVgp-CT6K/R), in which cytoplasmic lysine residues at positions 489/508/517 or 465/471/478/487/492/496 were mutated to arginine residues, was created by inserting overlapping PCR fragments into correspondingly digested pC-RABVg or pC-LCMVgp, respectively. Mutants of SARS-CoV-S and SARS-CoV-2-S (pC-SARS-S-CT4K/R, or pC-SARS2-S-CT4K/R), in which cytoplasmic lysine residues at positions 1227/1237/1248/1251 and 1245/1255/1266/1269 were mutated to arginine residues, were created by inserting overlapping PCR fragments into correspondingly digested pC-SARS-S and pC-SARS2-S, respectively. The CHIKV and RRV 6K mutants (pC-CHIKVe-6K-2K/R and pC-RRVe-6K-2K/R), in which the 6K region’s cytoplasmic lysine residues at positions 37/44 were mutated to arginine residues, were created by inserting overlapping PCR fragments into correspondingly digested pC-CHIKVe and pC-RRVe, respectively. Similarly, the E2 mutants of CHIKV and RRV (pC-CHIKVe-E2-K/R or pC-RRVe-E2-K/R), in which E2’s C-terminal lysine residues at positions 422 and 394 were mutated to arginine residues, were created by inserting overlapping PCR fragments into correspondingly digested pCAGGS. The C-terminally T7e–tagged wild-type and its mutant expression plasmids of RABV-G (pC-RABVg-T7e and pC-RABVg-CT3K/R-T7e), LCMV-GP (pC-LCMVgp-T7e and pC-LCMVgp-CT6K/R-T7e), SARS-CoV-S (pC-SARS-S-T7e and pC-SARS-S-CT4K/R-T7e), SARS-CoV-2-S (pC-SARS2-S-T7e and pC-SARS2-S-CT4K/R-T7e), CHIKV-E3-E2-6K-E1 (pC-CHIKVe-T7e, pC-CHIKVe-6K-2K/R-T7e, and pC-CHIKVe-E2-K/R-T7e), and RRV-E3-E2-6K-E1 (pC-RRVe-T7e, pC-RRVe-6K-2K/R-T7e, and pC-RRVe-E2-K/R-T7e) were created by inserting the corresponding PCR fragments into pCAGGS in which T7e along with a six-glycine linker was C-terminally tagged. To detect ubiquitinated CHIKV-E2 proteins by immunoprecipitation assays, WT or E2-K/R mutant plasmids expressing CHIKV E3-E2-6K-E1, in which the T7 epitope-tag is inserted immediately downstream of a furin-cleavage site between E3 and E2, were created by inserting overlapping PCR fragments that harbor a T7e sequence into correspondingly digested pCAGGS and were designated pC-CHIKVe-T7eE2 and pC-CHIKVe-T7eE2-K/R, respectively. All constructs were verified by a DNA sequencing service (FASMAC). Table S1 in the supplemental material contains additional information on oligonucleotides.

### Cells.

293T, HeLa, NIH 3T3, HOS, and M8166+MARCH8 (14) cells were maintained under standard conditions. Cells were originally obtained from ATCC (except for M8166+MARCH8 cells) and routinely tested negative for mycoplasma contamination (PCR Mycoplasma Detection Set, TaKaRa).

### Virion infectivity assay.

To prepare various viral envelope glycoprotein-pseudotyped HIV-1 luciferase reporter viruses, 1.1 × 10^5^ 293T cells were cotransfected with increasing amounts of the MARCH8 expression plasmid, 20 ng of viral envelope glycoprotein expression plasmid (pC-RABVg, pC-LCMVgp, pC-SARS-S, pC-SARS2-S, pC-CHIKVe, pC-RRVe, and their mutants), 500 ng of pNL-Luc2-IN/HiBiT-E(-)Fin, and an empty vector up to 1 μg of total DNA using FuGENE6. Sixteen hours later, transfected cells were washed with phosphate-buffered saline, and 1 ml of fresh complete medium was added. After 24 h, the supernatants were harvested and treated with 37.5 U/ml DNase I (Roche) at 37°C for 30 min. Viral supernatants were measured by the HiBiT assay, as previously described (19). Briefly, a standard virus stock with known levels of p24 antigen was serially diluted. Either the standards or viral supernatants containing pseudotyped viruses (25 μl) and LgBiT Protein (1:100)/HiBiT Lytic Substrate (1:50) in Nano-Glo HiBiT Lytic Buffer (25 μl) (Nano-Glo HiBiT Lytic Detection System; Promega) were mixed and incubated for 10 min at room temperature according to a modified version of the manufacturer's instructions. HiBiT-based luciferase activity in viral supernatants was determined with a Centro LB960 luminometer (Berthold) and was translated into p24 antigen levels. To determine viral infectivity, 1 × 10^4^ NIH 3T3 cells or HeLa cells were incubated with 1 ng of p24 antigen of either arenavirus virus envelope (LCMV-GP)- or alphavirus E3-E2-6K-E1 envelope (derived from CHIKV or RRV)-pseudotyped HIV-1 luciferase reporter viruses, respectively. Additionally, 2.2 × 10^4^ 293T cells were incubated with 1 ng of p24 antigen of HIV-1 luciferase reporter viruses pseudotyped with RABV-G. Alternatively, 2.2 × 10^4^ 293T cells transiently coexpressing ACE2 and TMPRSS2 (using pC-ACE2 and pC-TMPRSS2) were incubated with 1 ng of p24 antigen of either SARS-CoV-S- or SARS-CoV-2-S-pseudotyped luciferase reporter lentiviruses. After 48 h, cells were lysed in 100 μl of One-Glo luciferase assay reagent (Promega). The firefly luciferase activity was determined with a Centro LB960 (Berthold) luminometer and exported to Microsoft Excel 2016 through MicroWin version 4.36 (Mikrotek Laborsysteme).

### Immunoblotting assays.

Cells transfected as descried above were lysed in 75 μl of RIPA buffer (50 mM Tris-HCl, pH 7.4, 150 mM NaCl, 0.5% sodium deoxycholate, 1% Nonidet P-40, 0.1% SDS, and cOmplete Protease Inhibitor Mixture [Roche Applied Science]) and sonicated briefly on ice. Cell extracts were then subjected to gel electrophoresis and transferred to a PVDF membrane. The membranes were probed with an anti-T7 epitope mouse monoclonal antibody (1:3,000; Novagen, 69522-4), an anti-MARCH8 rabbit polyclonal antibody (1:360) (12), anti-p24 monoclonal antibody (1:1,000; Nu24 [14]), and an anti-β-actin mouse monoclonal antibody (1:5,000; Sigma-Aldrich, A5316). Viral supernatants containing were layered onto 20% (wt/vol) sucrose cushions and subjected to ultracentrifugation (90,000 r.p.m. for 10 min) using an Optima TLX Ultracentrifuge (Beckman Coulter). Pelleted virions were resuspended in SDS-sample buffer and subjected to Western blot analysis using the anti-T7 epitope mouse monoclonal antibody (1:3,000), the anti-MARCH8 rabbit polyclonal antibody (1:360), and the Nu24 anti-p24 monoclonal antibody (1:1,000). Reacted proteins were visualized by chemiluminescence using a Clarity Western ECL Substrate (Bio-Rad) and monitored using a LAS-3000 imaging system (FujiFilm).

### Immunofluorescence microscopy.

HOS cells were plated on 13-mm coverslips, cotransfected with 0.5 μg of pC-xx-T7e (xx: RABV-G, RABV-G-CT3K/R, LCMVgp, LCMVgp-CT6K/R, SARS-S, SARS-S-CT4K/R, SARS2-S, SARS2-S-CT4K/R, CHIKVe, CHIKVe-6K-2K/R, CHIKVe-E2-K/R, RRVe, RRVe-6K-2K/R, or RRVe-E2-K/R), 0.1 μg of pC-GagPol-RRE, 0.05 μg of pCa-Rev, and 0.3 μg of either the HA-MARCH8 expression plasmid or an empty control using FuGENE6, and cultured for 24 h. To examine whether WT envelope glycoproteins are lysosomally degraded by MARCH8, the transfected cells were cultured in the presence of lysosomal inhibitors (either 40 μM leupeptin and pepstatin A (Peptide Institute Inc., 4041 and 4397-v, respectively) or 5 μM bafilomycin A1 (Adipogen Life Sciences, BVT-0252)) for 14 h. For the total staining of both Env-T7es and HA-MARCH8, cells were fixed with 4% paraformaldehyde for 30 min on ice and permeabilized with 0.05% saponin. Fixed cells were incubated with anti T7 epitope mouse monoclonal antibodies (Novagen, 69522-4) and anti-HA rabbit polyclonal antibodies (Sigma-Aldrich, H6908). The secondary antibodies Alexa 488 donkey anti-mouse IgG (Molecular Probes, A-21202) and Alexa 568 donkey anti-rabbit IgG (Molecular Probes, A-10042) were used for double staining. Confocal images were obtained with a FluoView FV10i automated confocal laser-scanning microscope (Olympus) and analyzed with FluoView software version 3.1b (Olympus). For RABV whole-virus infection of cells stably expressing MARCH8, M8166+MARCH8 cells were plated on CELLview dishes (35-mm, 4-compartment cell culture dishes with a glass bottom; Greiner Bio-One), infected with the RABV CVS-26 strain (25) at a multiplicity of infection of 0.5, and cultured for 24 h. To detect cell-surface RABV-G protein, cells were fixed with 4% paraformaldehyde neutral buffer solution, pH 7.4 (Wako Pure Chemical, Osaka, Japan), at room temperature for 30 min. Fixed cells were incubated with an anti-RABV-G mouse monoclonal number 7-1-9 (0.4 mg/ml) (29). To detect intracellular RABV-P protein, cells were permeabilized with 0.05% Triton X-100 in PBS for 5 min at room temperature and incubated with anti-RABV-P rabbit polyclonal antibody (30). The secondary antibodies Alexa 488 donkey anti-mouse IgG and Alexa 568 donkey anti-rabbit IgG were used for staining. Quantitative analyses were conducted by counting positively stained cells in 10 randomly selected fields. Nuclei were visualized by DAPI (Sigma-Aldrich, D9542) staining. Confocal images were obtained with an FV1000 confocal laser scanning microscope (Olympus) and analyzed with FluoView software version 3.1b (Olympus).

### Ubiquitination assay.

293T cells (5 × 10^5^) were cotransfected with 1.2 μg of pC-RABVg-T7e, pC-RABVg-CT3K/R-T7e, pC-CHIKVe-T7eE2, or pC-CHIKVe-T7eE2-K/R; and 0.3 μg of pC-HA-MARCH8 or an empty control. After 48 h, cells were lysed in TBS-T buffer (50 mM Tris-HCl buffer [pH 7.5], 0.15 M NaCl, 1% Triton X-100, and 0.5% deoxycholic acid) containing protease inhibitor cocktail and 10 mM N-ethylmaleimide as an inhibitor of deubiquitination enzymes. The mixture was centrifuged at 21,500 × *g* for 15 min, and the supernatant was used as total cell lysate for immunoblotting or immunoprecipitation. Fifty microliters of protein A-coupled Sepharose 4B (GE Healthcare, 17-0780-01) was preincubated for 2 h at 4°C with 4 μg of anti-ubiquitin mouse monoclonal antibodies (Clone FK2, Cayman, 14220). Total cell lysate was incubated with antibody-coupled Sepharose for 20 h at 4°C. The Sepharose was washed three times with TBS-T buffer and one time with PBS before the immunoprecipitated proteins were eluted with SDS sample buffer. To evaluate ubiquitination states, proteins immunoprecipitated with anti-ubiquitin mouse antibodies were subjected to Western blotting with anti-T7e rabbit antibodies (MBL, PM022). Total cell lysate was also subjected to immunoblotting with anti-T7e rabbit antibodies and anti-HA rabbit polyclonal antibodies (Sigma-Aldrich, H6908) to evaluate the expression levels of Env-T7es and HA-MARCH8. Immunoreactive bands were detected using an ECL detection kit (ATTO, EzWestLumi plus, WSE-7120) with a ChemiDoc imaging system (Bio-Rad Laboratories). Images were then analyzed using Image Lab Software version 5.2.1 (Bio-Rad Laboratories).

### Statistical analysis.

Column graphs that combine bars and individual data points were created with GraphPad Prism version 9.10. *P values* generated from one-way analysis of variance and Dunnett’s multiple-comparison tests for data represented in Fig. 1 (C, E, G, H, J, and K), S3, S4, and S5, and from two-tailed unpaired t-tests for data represented in Fig. 2 (B, E, H, and K), and 3 (B, D, G, and I).

### Data availability.

All data generated or analyzed during this study are included in this article and the supplemental material. Constructs are available via requests to the corresponding author.
