# Supplementary material for: MARCH8 Targets Cytoplasmic Lysine Residues of Various Viral Envelope Glycoproteins
Source: Microbiol Spectr. 2022 Jan 12;10(1):e00618-21. doi: 10.1128/spectrum.00618-21 (PMC8754143; doi:10.1128/spectrum.00618-21)
Supplement: SUPPLEMENTAL FILE 1 — Supplemental material. Download SPECTRUM00618-21_Supp_1_seq8.pdf, PDF file, 1 MB [file spectrum00618-21_supp_1_seq8.pdf]

Supplementary information

MARCH8 targets cytoplasmic lysine residues of various viral envelope glycoproteins

Yanzhao Zhang, Seiya Ozono, Takuya Tada, Minoru Tobiume, Masanori Kameoka,
Satoshi Kishigami, Hideaki Fujita, and Kenzo Tokunaga

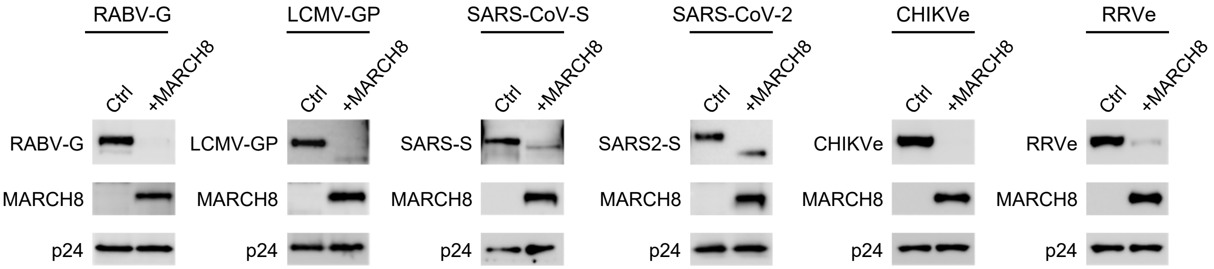

Figure S1. MARCH8-mediated reduction of virion incorporation of various viral envelope glycoproteins. Virus particles were concentrated through a sucrose cushion by ultracentrifugation of the viral supernatants of pseudotyped viruses produced from cells expressing MARCH8 or the vector control, and subjected to Western blot analysis. Antibodies specific for T7 epitope tag to detect each viral envelope glycoproteins (upper), MARCH8 (middle), and p24 (lower) were used. Data shown are representative of two independent experiments.

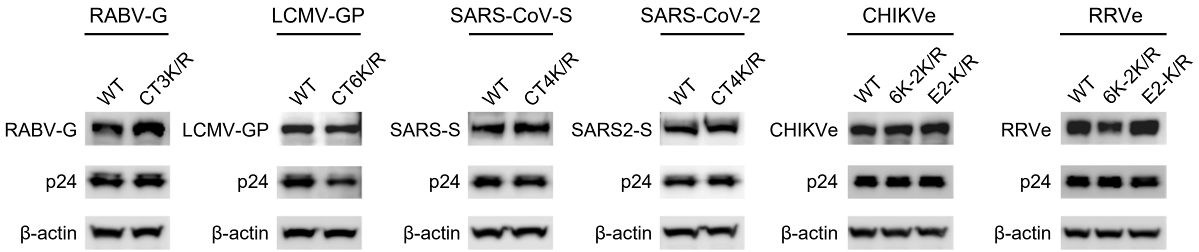

Figure S2. Expression of WT and mutant envelope glycoproteins. Western blot analysis performed by using lysates from 293T cells producing viruses pseudotyped with various viral envelope glycoproteins. Antibodies specific for T7 epitope tag to detect each viral envelope glycoproteins (upper), p24 (middle), and β-actin (lower) were used. Data shown are representative of two independent experiments.

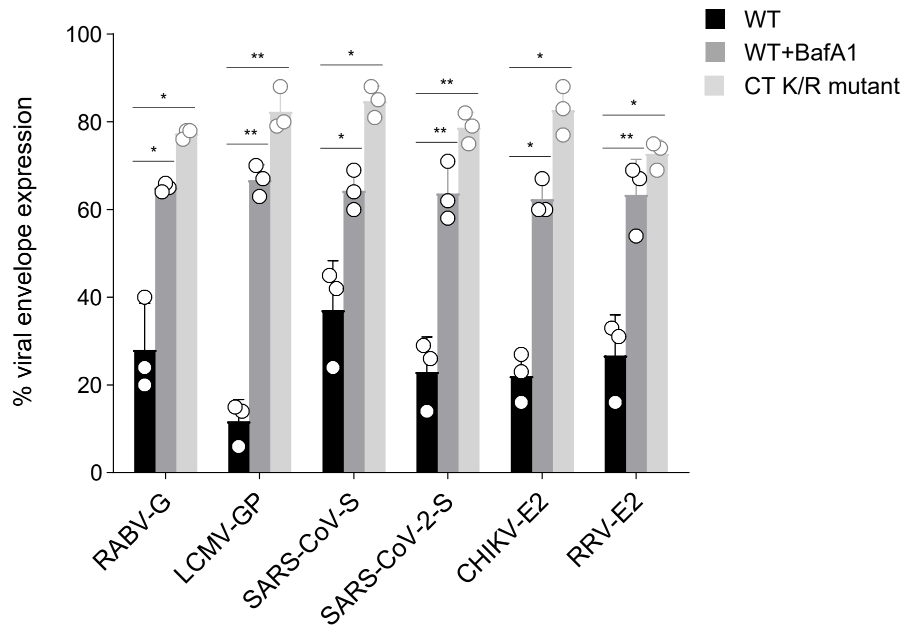

Figure S3. MARCH8-induced degradation of various viral envelope glycoproteins is rescued by bafilomycin A1 or by lysine mutation(s) in their cytoplasmic tails. Using immunofluorescence data obtained in the experiments of Figs 2 and 3, quantitative analyses were performed by counting cells expressing viral envelope glycoproteins in MARCH8-positive cells. Data are shown as a percentage of viral envelope expression in cells expressing MARCH8 (mean + s.d. from three independent experiments). **p*<0.05, ***p*<0.005, compared with WT using one-way analysis of variance and Dunnett's multiple comparison tests. WT (black), cells expressing WT viral envelope glycoproteins; WT+BafA1 (dark grey), cells expressing WT in the presence of bafilomycin A1 (5 µM); CT K/R mutant (light grey), cells expressing their cytoplasmic lysine mutants.

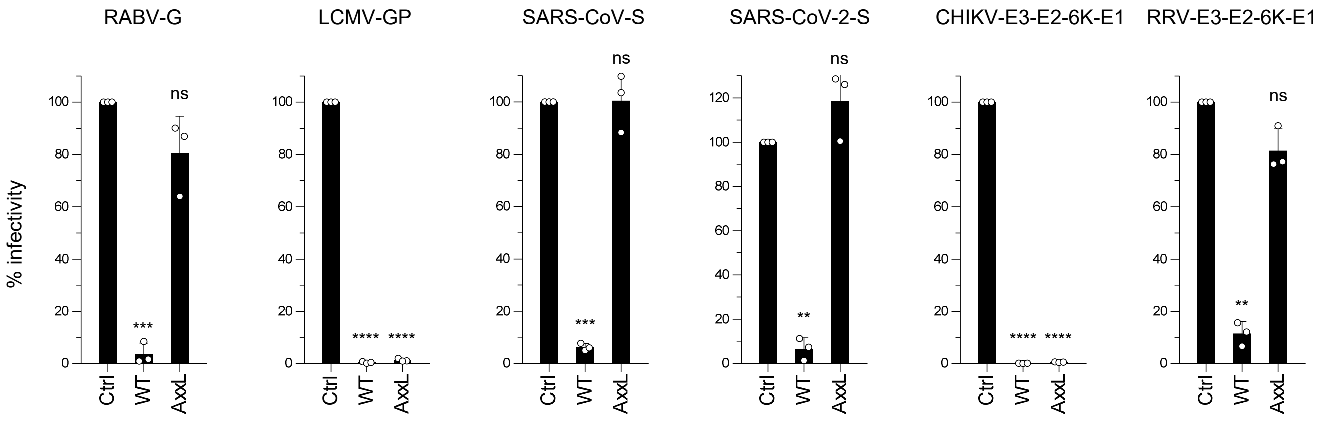

Figure S4. The tyrosine motif of MARCH8 partially mediates the downregulation of RABV-G, SARS-CoV-S, SARS-CoV-2-S, and RRV-E3-E2-6K-E1 but not LCMV-GP or CHIKV-E3-E2-6K-E1. Infectivity of viruses prepared from 293T cells cotransfected with Env-defective HiBiT-tagged HIV-1 luciferase (luc) reporter proviral DNA clone pNL-Luc2-IN/HiBiT-E(-)Fin and a control (Ctrl), WT, or ^{222}AxxL^{225} MARCH8 plasmid, together with an RABV-G, LCMV-GP, SARS-CoV-S, SARS-CoV-2-S, CHIKV-E3-E2-6K-E1, or RABV-E3-E2-6K-E1 expression plasmid. Data are shown as a percentage of the viral infectivity in the absence of MARCH8 (mean + s.d. from three independent experiments). **p*<0.05, ***p*<0.005, ****p*<0.0005, *****p*<0.0001 compared with the Ctrl using one-way analysis of variance and Dunnett's multiple comparison tests. ns, not significant.

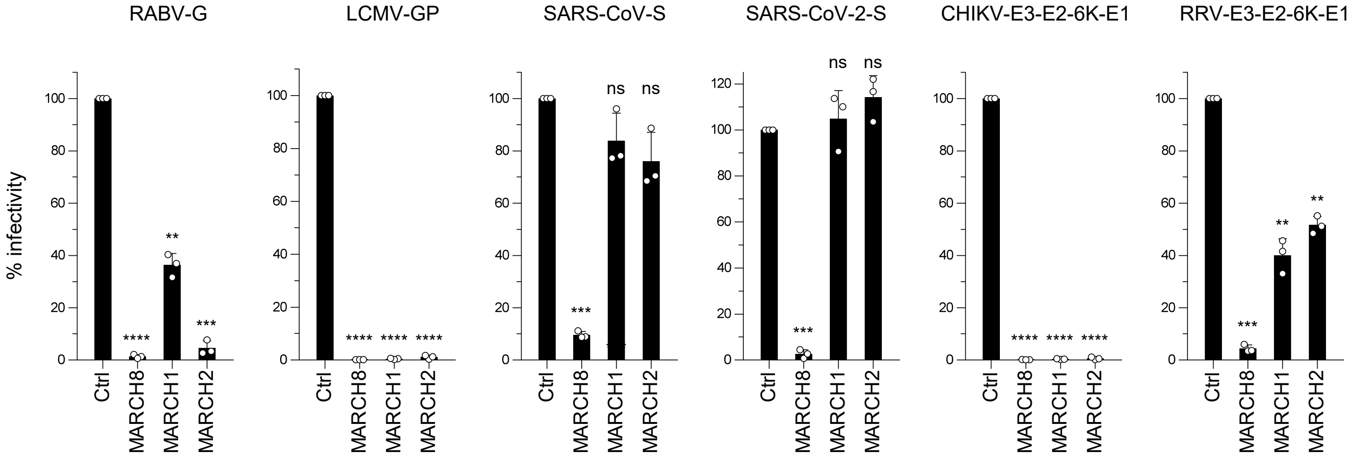

Figure S5. MARCH1 and MARCH2 differentially target a variety of viral envelope proteins. Infectivity of viruses prepared from 293T cells cotransfected with Env-defective HiBiT-tagged HIV-1 luciferase (luc) reporter proviral DNA clone pNL-Luc2-IN/HiBiT-E(-)Fin and a control (Ctrl), MARCH1, MARCH2, or MARCH8 plasmid, together with an RABV-G, LCMV-GP, SARS-CoV-S, SARS-CoV-2-S, CHIKV-E3-E2-6K-E1, or RABV-E3-E2-6K-E1 expression plasmid. Data are shown as a percentage of the viral infectivity in the absence of MARCH8 (mean + s.d. from three independent experiments). *p<0.05, **p<0.005, ***p<0.0005, ****p<0.0001 compared with the Ctrl using one-way analysis of variance and Dunnett's multiple comparison tests. ns, not significant.

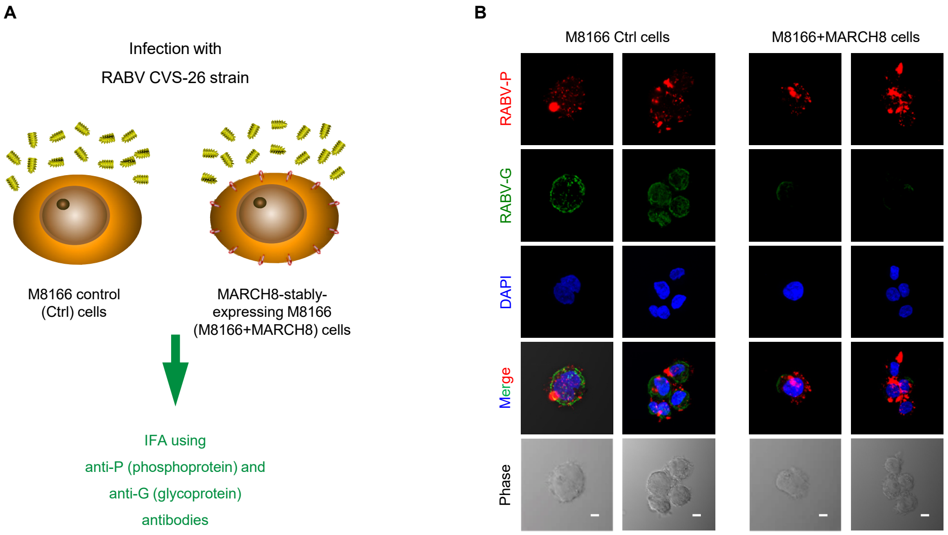

Figure S6. Antiviral activity of MARCH8 is reproducible in whole-virus-based infection. (**A**) Schematic flowchart of the infection of MARCH8-stably expressing M8166+MARCH8 cells with the RABV CVS-26 strain. (**B**) MARCH8 downregulates the G protein of the RABV CVS-26 strain. Shown are immunofluorescence images of the cell-surface expression of RABV-G and the intracellular expression of RABV phosphoprotein P (RABV-P) in M8166 control (Ctrl) cells (left panels) and M8166+MARCH8 cells (right panels). Scale bars, 10 µm.

NL4-3-gp41: ^{704}NRVRQGYSPLSFQTHLPIPRGPDRPEGIEEEGGERDRDRSIRLVNGSLALIWDDLRSLCLFSYHRLRD
LLLIVTRIVELLGRRGWEALKYWWNLLQYWSQELKNSAVNLLNATAIAVAEGTDRVIEVLQAAYRAIRHI
PRRIRQGLERILL^{854}

VSV-G: ^{483}RVGIHLCIKLKHTKKRQIYTDIEMNRLGK^{511}

RABV-G: ^{481}RRANRPESKQRSFGGTGGNVSVTSQSGKVIPSWESYKSGGEIRL^{524}

LCMV-GP: ^{460}THRHIKGGSCPKPHRLTNKGICSCGAFKVPGVKTVWKRR^{498}

SARS-CoV-S: ^{1217}CCMTSCCSCLKGACSCGSCCKFDEDDSEPVLKGVKLHYT^{1255}

SARS-CoV2-S: ^{1235}CCMTSCCSCLKGCCSCGSCCKFDEDDSEPVLKGVKLHYT^{1273}

CHIKV-E2: ^{391}CARRRCITPYELTPGATVPFLLSLLCCVRTTKA^{423}

RRV-E2: ^{390}TARRKCLTPYALTPGAVVPLTLGLLCCAPRANA^{422}

Figure S7. Amino acid sequences of the cytoplasmic tails of the viral envelope glycoproteins tested in our previous and current studies. Among cytoplasmic tail sequences shown above, the alphavirus E2 proteins (CHIKV-E2 and RRV-E2) contain the second transmembrane domains, which are released from the membrane after its cleavage and is then translocated (along with its short ectodomain) into the cytoplasm.

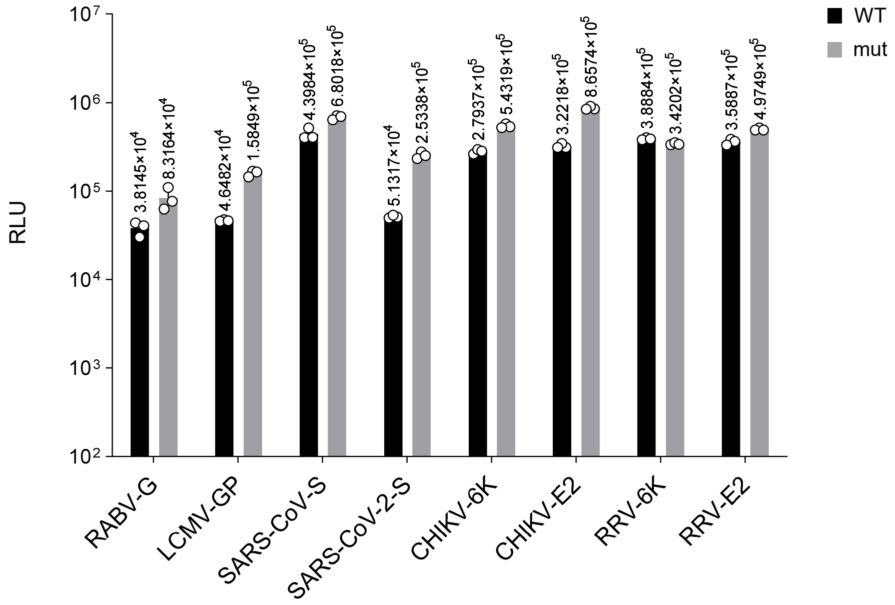

Figure S8. Firefly luciferase values before individual normalization. Shown are the firefly luciferase values obtained in infection of different target cells with the indicated wild-type (WT; black) or mutant (mut; grey) pseudoviruses produced from transfected 293T cells in the absence of MARCH8. Representative data from three independent experiments are shown. RLU, relative light units.

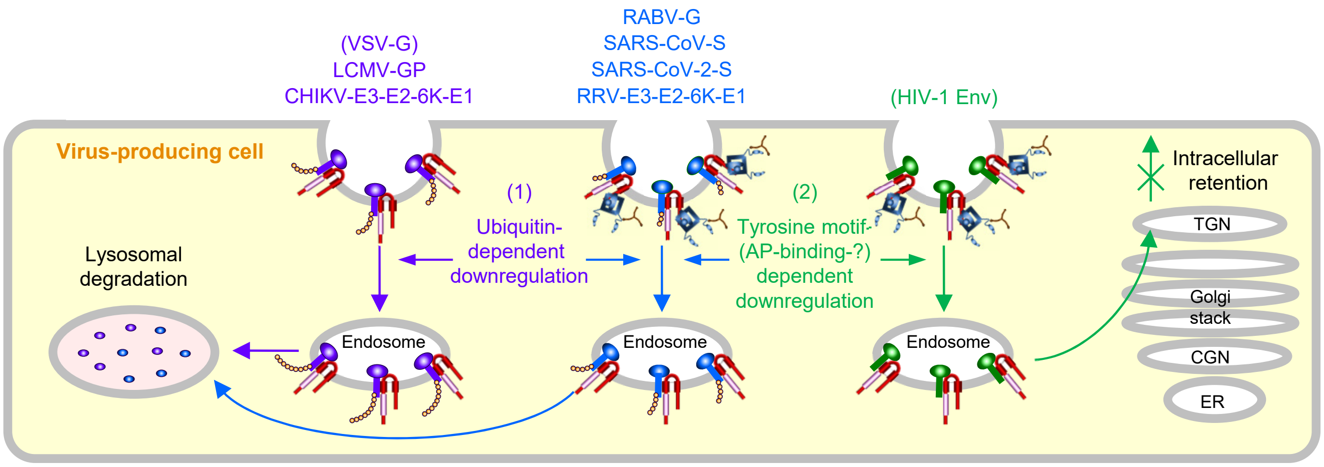

Figure S9. Schematic diagram of different mechanisms by which MARCH8 downregulates viral envelopes. *Left*, MARCH8 (red) downregulates VSV-G, LCMV-GP, and CHIKV-E3-E2-6K-E1 (violet) in a ubiquitin-dependent manner. The RING-CH domain (pink) of MARCH8 mediates ubiquitin conjugation (shown as orange beads) at cytoplasmic lysine residue(s) of VSV-G, LCMV-GP and CHIKV-E2, leading to lysosomal degradation. *Right*, MARCH8 downregulates HIV-1 Env (green) in a tyrosine motif-dependent manner, as previously described (1). The tyrosine motif in the C-terminal CT of MARCH8 likely interacts with the adaptor protein μ-subunit (navy) (involving clathrin (brown) if this is the case with μ2 or μ1), resulting in the TGN retention of HIV-1 Env. *Middle*. MARCH8 downregulates RABV-G, SARS-CoV-S, SARS-CoV-2-S, and RRV-E3-E2-6K-E1 (blue) in both ubiquitin- and tyrosine motif-dependent manners, leading to lysosomal degradation. Note that MARCH8-induced downregulation of these viral glycoproteins might not necessarily occur at the plasma membrane. The nucleus and other organelles are not shown. The image was adapted from that of Zhang *et al*. (1).

REFERENCES

1. Zhang Y, Tada T, Ozono S, Kishigami S, Fujita H, Tokunaga K. 2020. MARCH8 inhibits viral infection by two different mechanisms. Elife 9:e57763.

TABLES1: Primers used in this study

Designation	Source	Identifiers	Additional information
RABVg-Acc-S	This paper	ggGGTACCatggttcctcaggttcttttg	Sense primer for pC-RABVg, pC-RABVg-CT3K/R, pC-RABVg-T7e, and pC-RABVg-CT3K/R-T7e
RABVg-Xho-A	This paper	ccgCTCGAGtcacagtctgatctcacctc	Antisense primer for pC-RABVg, and pC-RABVg-CT3K/R
LCMVgp-BsiW-S	This paper	atCGTACGatgggtcagattgtgacaatg	Sense primer for pC-LCMVgp, pC-LCMVgp-CT6K/R, pC-LCMVgp-T7e, and pC-LCMVgp-CT6K/R-T7e
LCMVgp-Xho-A	This paper	ccgCTCGAGtcagcgtcttttccagacggt	Antisense primer for pC-LCMVgp
CHIKVe-Acc-S	This paper	ggGGTACCatgagtctggccctgccagtg	Sense primer for pC-CHIKVe, pC-CHIKVe-6K-2K/R, pC-CHIKVe-E2-K/R, pC-CHIKVe-T7e, pC-CHIKVe-6K-2K/R-T7e, pC-CHIKVe-E2-K/R-T7e, pC-CHIKVe-T7eE2, and pC-CHIKVe-T7eE2-K/R
CHIKVe-Xho-A	This paper	ccgCTCGAGtcaatgcctggaaaatgagac	Antisense primer for pC-CHIKVe, pC-CHIKVe-6K-2K/R, pC-CHIKVe-E2-K/R, pC-CHIKVe-T7eE2, and pC-CHIKVe-T7eE2-K/R
RRVe-EcoV-S	This paper	ccgGATATCatgtctgccgcgctgatgatgtg	Sense primer for pC-RRVe, pC-RRVe-6K-2K/R, pC-RRVe-E2-K/R, pC-RRVe-T7e, pC-RRVe-6K-2K/R-T7e, and pC-RRVe-E2-K/R-T7e
RRVe-Not-A	This paper	attGCGGCCGCttaccgacgcattgttatgc	Antisense primer for pC-RRVe, pC-RRVe-6K-2K/R, and pC-RRVe-E2-K/R
RABVg-CT3K/R-S1	This paper	gaatcTaGacaacgcagttttggagggaca	1st round overlapping PCR's sense primer for pC-RABVg-CT3K/R
RABVg-CT3K/R-A1	This paper	tgtccctccaaaactgcgttgtCtAgattc	1st round overlapping PCR's antisense primer for pC-RABVg-CT3K/R
RABVg-CT3K/R-S2	This paper	caaagcggaaGagtcataccttcatgggaatcatataGgagt	2nd round overlapping PCR's sense primer for pC-RABVg-CT3K/R
RABVg-CT3K/R-A2	This paper	actcCtatatgattcccatgaaggtatgactCttccgctttg	2nd round overlapping PCR's antisense primer for pC-RABVg-CT3K/R
LCMVgp-CT6K/R-S	This paper	cacataaGaggtggctcatgtccaaGgccacaTcgattaacca acaGaggaatttgtc	Overlapping PCR's sense primer for the 3' fragment of pC-LCMVgp-CT6K/R
LCMVgp-CT6K/R-A	This paper	acaaattcctCtgttggttaatcgAtgtggcCttggacatgagcca cctCttatgtg	Overlapping PCR's antisense primer for the 5' fragment of pC-LCMVgp-CT6K/R
LCMVgp-CT6K/R-Xho-A	This paper	ccgCTCGAGtcagcgtcttCtccagacggttCttacaccagg caccCtaaatgc	Overlapping PCR's antisense primer for the 3' fragment of pC-LCMVgp-CT6K/R
SARS-S-BsiW-S	Ozono et al. 2021	atCGTACGccatgttcatcttcctgctgttcct	Sense primer for pC-SARS-S-CT4K/R, pC-SARS-S-T7e, and pCS-CT4K/R-S-T7e
SARS-S-CT4K/R-S	This paper	gcAtgcagctgcggcagctgctgcaGgttcgacgagga	Overlapping PCR's sense primer for the 3' fragment of pC-SARS-S-CT4K/R
SARS-S-CT4K/R-A	This paper	cagctgccgcagctgcaTgcgcccCtcaggcag	Overlapping PCR's antisense primer for the 5' fragment of pC-SARS-S-CT4K/R
SARS-S-CT4K/R-Xho-A	This paper	ccgCTCGAGttaggtgtagtgcagtCtcactcccCtcagcac agg	Overlapping PCR's antisense primer for the 3' fragment of pC-SARS-S-CT4K/R
SARS2-S-Acc-S	Ozono et al. 2021	ggGGTACCatgtttgtgttcctggtgct	Sense primer for pC-SARS2-S-CT4K/R, pC-SARS2-S-T7e, and pC-SARS2-S-CT4K/R-S-T7e
SARS2-S-CT4K/R-S	This paper	tgttgttcctgtggAtcctgttgtaGgtttgatgagga	Overlapping PCR's sense primer for the 3' fragment of pC-SARS2-S-CT4K/R
SARS2-S-CT4K/R-A	This paper	caggaTccacaggaacaacagcctCtcagacaggaac	Overlapping PCR's antisense primer for the 5' fragment of pC-SARS2-S-CT4K/R
SARS2-S-CT4K/R-Not-A	This paper	atatGCGGCCGCtcaggtgtagtgcagtCtcactcctCtcag cac	Overlapping PCR's antisense primer for the 3' fragment of pC-SARS2-S-CT4K/R
CHIKVe-6K-2K/R-S	This paper	aactgcctgaGgctgctgccatgctgttgcaGaacactggca	Overlapping PCR's sense primer for the 3' fragment of pC-CHIKVe-6K-2K/R
CHIKVe-6K-2K/R-A	This paper	tgccagtgttCtgcaacagcatggcagcagcCtcaggcagtt	Overlapping PCR's antisense primer for the 5' fragment of pC-CHIKVe-6K-2K/R
RRVe-6K-2K/R-S	This paper	catgctgtatcaGaTCTctgatctgctgttgtaGgccattttct	Overlapping PCR's sense primer for the 3' fragment of pC-RRVe-6K-2K/R
RRVe-6K-2K/R-A	This paper	agaaaatggcCtacaacagcagatcagAGAtCtgatacagc atg	Overlapping PCR's antisense primer for the 5' fragment of pC-RRVe-6K-2K/R
CHIKVe-E2-K/R-S	This paper	gtgcggactaccaGggctgcaacc	Overlapping PCR's sense primer for the 3' fragment of pC-CHIKVe-E2-K/R
CHIKVe-E2-K/R-A	This paper	ggttgcagccCtggtagtccgcac	Overlapping PCR's antisense primer for the 5' fragment of pC-CHIKVe-E2-K/R
RRVe-E2-K/R-S	This paper	ccgcgaggagaaGgtgcctaacacc	Overlapping PCR's sense primer for the 3' fragment of pC-RRVe-E2-K/R
RRVe-E2-K/R-A	This paper	ggtgttaggcacCttctcctcgcgg	Overlapping PCR's antisense primer for the 5' fragment of pC-RRVe-E2-K/R
Xho-6G-T7e-Not-S	This paper	tcgagggtggcggaggaggtggcatggcttctatgactggtggtc aacaaatgggttagc	Sense linker (XhoI/NotI) for six-glycine (6G) plus T7 epitope (T7e) tag
Xho-6G-T7e-Not-A	This paper	ggccgctaacccatttgttgaccaccagtcatagaagccatgcca cctcctccgccaccc	Antisense linker (XhoI/NotI) for six-glycine (6G) plus T7 epitope (T7e) tag
Not-6G-T7e-Sac-S	This paper	ggccgccggtggcggaggaggtggcatggcttctatgactggtg gtcaacaaatgggttaagct	Sense linker (NotI/SacI) for six-glycine (6G) plus T7 epitope (T7e) tag
Not-6G-T7e-Sac-A	This paper	taacccatttgttgaccaccagtcatagaagccatgccacctcctc cgccaccggc	Antisense linker (NotI/SacI) for six-glycine (6G) plus T7 epitope (T7e) tag
RABVg-fs-Xho-A	This paper	ccgCTCGAGcagtctgatctcacctccac	Antisense primer for pC-RABVg-T7e, and pC-RABVg-CT3K/R-T7e
LCMVgp-fs-Xho-A	This paper	ccgCTCGAGgcgtcttttccagacggttt	Antisense primer for pC-LCMVgp-T7e
LCMVgp-CT6K/R-fs-Xho-A	This paper	ccgCTCGAGgcgtcttCtccagacggttc	Antisense primer for pC-LCMVgp-CT6K/R-T7e
SARS1/2-S-Xho-A	This paper	ccgCTCGAGggtgtagtgcagtttcactc	Antisense primer for pC-SARS-S-T7e, and pC-SARS2-S-T7e
SARS1/2-S-CT4K/R-Xho-A	This paper	ccgCTCGAGggtgtagtgcagtctcactc	Antisense primer for pC-SARS-S-CT4K/R-T7e, and pC-SARS2-S-CT4K/R-T7e
CHIKVe-fs-Xho-A	This paper	ccgCTCGAGatgcctggaaaatgagacgc	Antisense primer for pC-CHIKVe-T7e, pC-CHIKVe-6K-2K/R-T7e, and pC-CHIKVe-E2-K/R-T7e
RRVe-fs-Not-A	This paper	atatGCGGCCGCccgacgcattgttatgcagg	Antisense primer for pC-RRVe-T7e, pC-RRVe-6K-2K/R-T7e, and pC-RRVe-E2-K/R-T7e
CHIKVe-T7eE2-S	This paper	ATGGCTTCTATGACTGGTGGTCAACAAATGG GTggtggctccacaaaggacaacttcaac	Overlapping PCR's sense primer for the 3' fragment of pC-CHIKVe-T7eE2 and pC-CHIKVe-T7eE2-K/R
CHIKVe-T7eE2-A	This paper	GCCACCACCCATTTGTTGACCACCAGTCATA GAAGCCATccgtcgctggcgatgtggtga	Overlapping PCR's antisense primer for the 5' fragment of pC-CHIKVe-T7eE2 and pC-CHIKVe-T7eE2-K/R
